# Applying deductive reasoning and the principles of particle physics to aging research

**DOI:** 10.18632/aging.203555

**Published:** 2021-09-20

**Authors:** Alibek Moldakozhayev, Albina Tskhay, Vadim N. Gladyshev

**Affiliations:** 1 Department of Neurology and Neurosurgery, McGill University, Montreal, Quebec H3A 2B4, Canada; 2 Metabolic Disorders and Complications Program, Research Institute of the McGill University Health Centre, Montreal, Quebec H4A 3J1, Canada; 3 Brain Repair and Integrative Neuroscience Program, Research Institute of the McGill University Health Centre, Montreal, Quebec H4A 3J1, Canada; 4 Department of Family Medicine, McGill University, Montreal, Quebec H3S 1Z1, Canada; 5 Division of Genetics, Department of Medicine, Brigham and Women's Hospital and Harvard Medical School, Boston, MA 02115, USA

**Keywords:** aging, damage, framework, particle physics, theory

## Abstract

Aging is debatably one of the biggest mysteries for humanity, a process consisting of myriads of genetic, molecular, environmental, and stochastic deleterious events, leading to a progressive loss of organism functionality. Aging research currently lacks a common conceptual framework, and one challenge in establishing it is the fact that aging is a highly complex process. To help develop a framework of standard aging rules, we suggest the use of deductive reasoning based on particle physics' principles. Specifically, the principles that we suggest applying to study aging are discreteness of processes, transformation as a result of interaction, and understanding of threshold. Using this framework, biological aging may be described as a sequence of highly discrete molecular transformations caused by a combination of various specific internal and external factors. Internal organismal function and interaction of an organism with the environment result in chronic accumulation of molecular damage and other deleterious consequences of metabolism and the consequent loss of system's functionality. The loss of functionality occurs as a series of thresholds the organism reaches before it turns into an utterly non-functional state. We discuss how having a common ground may benefit aging research, introduce the logic of new principles and analyze specific examples of how this framework could be used to study aging and design longevity interventions.

## INTRODUCTION

Aging is a term used to describe a process consisting of myriads of genetic, molecular, environmental, and stochastic deleterious events, leading to a progressive loss of functionality. Aging research is a multidimensional and multidisciplinary field; this complexity makes it difficult for those studying this process to have common ground, even when discussing the most fundamental aspects of aging. Despite being a subject of numerous studies over the last two centuries, there is currently no consensus on the nature of aging, e.g., there is a lack of agreement on whether aging is programmed, what its main mechanisms are, how it is related to mortality rate, functional decline and damage accumulation, when it begins, and how it can be managed [1]. Lack of a shared paradigm, which is relatively uncommon in natural sciences, makes the field organized and more fragmented [2]. In essence, it lacks a foundation on which various researchers can build the knowledge base for the field.

There was an attempt to establish aging axioms that would play a role in the aging paradigm [2]. However, being proposed almost a decade ago, these principles did not integrate well into aging research. This could be due to the possible lack of several essential features that would allow us to understand aging better from the perspective of natural science.

There are two ways to consider aging and any other process: deductive and inductive reasoning.

Most theories on aging are based on inductive reasoning, i.e., offer a transition from specific observations to generalized principles of aging. In this article, we attempt to apply deductive reasoning, i.e., to consider the process of biological aging through the prism of common knowledge about natural phenomena borrowed from particle physics.

### Aging theories

Several dozens of heterogenic aging theories exist, and the most prominent ones are summarized below:

- The programmed theory of aging posits that aging of organisms is beneficial as it frees resources used by older organisms for the next generations [3].
- The hyperfunction theory of aging states that aging results from hyperactivity of genes following the completion of development [4].
- Mutation accumulation and antagonistic pleiotropy evolutionary theories of aging posit that some mutations or alleles that cause damage later in life are either neutral and beneficial during the early-life period of active reproduction and therefore cannot be removed by negative selection [5–8].
- The free radical theory of aging considers reactive oxygen species, a by-product of metabolism, to be the primary cellular damage source [9]. Accumulation of this damage, in turn, according to the theory, leads to aging.
- Single damage type-based aging theories, such as telomere shortening theory, immune senescence theory, mitochondrial dysfunction theory, etc [1, 10].
- The disposable soma theory explains aging as an inability of organisms to simultaneously support maintenance and reproduction due to the insufficiency of resources [11].
- The reliability theory explains aging as a consequence of a systems redundancy, i.e. lack of irreplaceable elements in a system [12].

These theories, while having merit, touch upon different aspects of aging, and as such cannot be reconciled with one another. The deleteriome model attempted to combine and integrate the aspects of aging considered by other theories. It proposed that biological systems and processes are imperfect, and that metabolism inevitably leads to the generation of molecular damage and the myriad other deleterious consequences. The deleteriome is the term to describe cumulative molecular damage as well as other deleterious consequences of imperfect activities of biological systems affected by genetic, environmental, and stochastic processes at all levels and eventually leading to the loss of organism's functionality as it ages [13].

Some additional integrative notions that were offered to summarize and describe the types of cumulative damage observed in ageing are seven major damage types [14], the nine hallmarks [15], and the seven pillars [16]. The seven major damage types suggested by the authors to be the main targets of anti-ageing research are extracellular aggregates, death-resistant cells, extracellular matrix stiffening, intracellular aggregates, mitochondrial mutations, cancerous cells, and cell loss, or tissue atrophy [14]. The nine hallmarks include instability of the genome, telomere exhaustion, epigenetic changes, proteostasis loss, alteration of nutrient sensing, mitochondrial dysfunction, cellular senescence, stem cell shortage, and aberrated intercellular communication [15]. The seven pillars concept is comprised of macromolecular damage, epigenetic changes, chronic inflammation, loss of adaptation to stress conditions, proteostasis loss, stem cell shortage, and metabolism alteration [16].

We examined the theories, concepts and models mentioned above and applied deductive reasoning based on particle physics’ principles to begin developing a framework of standard aging rules, which would be in line with experiments and observations.

### The standard model of particle physics

There are two classes of elementary particles, called fermions and bosons. Interactions between fermions happen through four fundamental forces: gravitational, electromagnetic, strong nuclear, and weak nuclear. These forces are thought to represent forms of interaction that are non-reducible any further. While a curvature of spacetime represents a gravitational field, the other three forces can be mathematically described as discrete quantum fields carried by bosons as per Standard Model of particle physics [17–19]. The Higgs field, existing in every region of our Universe, is responsible for the fifth interaction, resulting in a gain of mass by particles interacting with the field (Table 1).

**Table 1:** Types of elementary particles, particles-carriers of interaction, and interaction.

**Fermions**	**Particles of spacetime**
*Quarks*	Up, charm, top
*(form protons and neutrons)*	Down, strange, bottom
*Leptons*	Electron, muon, tau
	Electron neutrino, muon neutrino, tau neutrino
**Bosons**	**Interaction/force carriers (messenger particles)**
	Gluon – strong nuclear force-carrier
	Photon – electromagnetic force-carrier
	Z boson – weak nuclear force-carrier
	W boson – weak nuclear force-carrier
	Higgs boson - responsible for the emergence of mass

### Interaction-transformation principle

### Every interaction leads to a transformation of all interacting subjects

**Figure 1:**
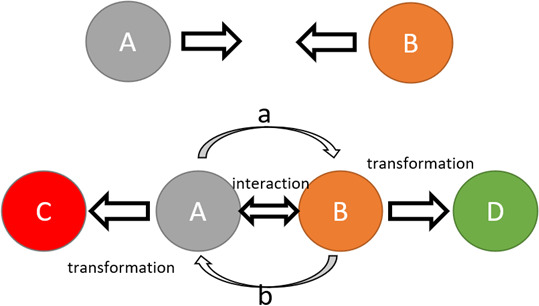
**Schematic representation of an interaction-transformation principle.** Upper case letters represent objects; lower case letters represent interaction/force carriers. Transformation of objects (A to C, B to D) happens following the interaction driven by carriers (a and b). The interaction results in a change of the physical state of the object. Carrier “a” of object “A”, carrying a part of the physical state of object “A” now becomes a part of object “B”, leading to the transformation of object “B” into object “D” and the other way around.

Fundamentally, interactions between fermions happen by boson exchange, and as a result, interacting fermions inevitably undergo transformations. Transformations of composite fermionic particles happen due to the exchange of energy, momentum and, in some cases, charge carried by bosons. The transformation of particles at a microscale eventually results in changes on a macroscale. The way transformations happen on a macroscale follows the same logic of the particle-level interaction-transformation principle. During an intermolecular interaction, molecules exchange physical characteristics, which results in attraction, repulsion, or bond formation, and a consequent physical transformation of the molecules. When cells interact with each other they exchange mediators using vesicles or junctions, which cause transformations of cells’ state. When people interact, they exchange physical force, or information, which leads to transformations of their physical and mental states [17–19]. Schematic representation of this principle is shown in Figure 1.

### Discreteness principle

### Every process can be dissociated from the chronological time and considered as a sequence of discrete events. It is the order and the number of these events that predetermines the outcome, not time

Continuity of the world is a mathematical method to describe what is made up of discrete grains on every scale [20]. Humans have organs, and organs are made of cells, while cells are made up of different organelles and molecular aggregates interacting with one another. Molecules are made from atoms, atoms are made of subatomic particles, and so on. Discreteness means that any process can be studied as a sequence of smaller steps. The idea of discreteness in aging was supported by Botwinick who stated that time is just an index of event occurring, and the time-dependent causality observed is in fact a causality between these events measured by time [21]. An excellent example of biological discreteness and the possibility to dissociate chronological time from aging was described by Hayflick, who showed that cells could replicate only a limited number of times, meaning that a cell’s age depends not on time per se but rather on the number of replication events preceding the moment of observation [22].

The principle of discreteness combined with the interaction-transformation principle suggests that any biological phenomenon, including aging, can be considered as a sequence of discrete interactions leading to observed transformation. Consequently, according to the proposed model, any given biological problem can potentially be solved either by preventing or inducing a sequence of specific interactions.

### The threshold principle

### The threshold value is predetermined by a probability for a specific interaction to cause a specific transformation. An at-the-threshold event occurs not because of the accumulation of prior stimuli but because a larger number of stimuli increased the probability of the observed event

In a search of these specific interactions, one can find that a relatively small fraction of atoms regulates transformations within the living matter. These atoms are the building blocks of DNA, RNA, and proteins. They are highly ordered, and the dislocation of just a few atoms within the group leads to the establishment of an alternative configuration having a different functionality. Both the original and final alternative configurations of these highly ordered molecules are generally energetically stable, meaning that spontaneous transition between the configurations is not possible [23]. Transformation of a system does not happen immediately – it occurs after the system reaches a certain threshold. However, in particle physics, the principle of a threshold is not considered – events either happen or they do not. It means that the notion of threshold should be considered discretely as well, as its perceived linearity is not confirmed on the particle level. Therefore, we define the threshold for a transformation as a point at which a discrete interaction leading to an alternative configuration occurs. Since the threshold, as well as all particle-level events, has a probabilistic nature, it can be described by a probability density function.

Therefore, a transformation leading to the change of functionality due to the damage caused by the interaction occurs not because there is too much damage in a system, but because too much damage in a system increases the probability of specific deleterious transformation to happen.

For example, a gene that maintains a critical functionality for a cell has a non-zero probability of being transformed (damaged). While damage may be produced by numerous processes, a combination of these damaging events will be considered up-to threshold events for that gene. Most likely happening precisely at that gene's structure, one specific damage could become a threshold leading to functionality alteration for that specific gene.

The variability of a threshold value for a transformation of a macroscale-level unit would depend on the variability of the characters of microscale-level interactions that can lead to state alteration. For example, a neuron typically needs to reach a threshold value between -50 and -55 mV to transform into an active state. At the same time, different neurons may have different threshold values depending on conductance and density of ion channels [24], as well as on environment-dependent factors such as amplitude and frequency of a signal on a certain plane, as in the example of a threshold for informing a brain about the displacement of a signal [25]. It was additionally shown that neurons possess threshold-driven plasticity in their functionality, meaning that the transformation of a neuron into an active state at different thresholds will result in differences in encoded information [26]. Importantly, it was confirmed that physical probability of a certain discrete interaction determines the level of a threshold for a response activation. Specifically, a higher threshold value during the stress response is explained by the reduced ability of the cellular machinery to activate the main heat shock-stress factor, HSF1 [27]. In this case, the threshold value is represented by a neuron's probability to activate an HSF1, which is further needed to promote stress response. Moreover, it was shown that a neuron is not a single excitable threshold unit – it instead consists of a number of discrete anisotropic threshold elements, which have certain threshold values only for certain incoming signals from a certain confined direction with no direct spatial summation [28]. In simple terms, for a standard neuron with a threshold value at -55 mV it would mean that the probability that transformation happens is close to 100% at a value of -55 mV. It does not mean that no action potential is impossible at lower values such as -65 mV; it still could happen, but far more rarely, since the probability is significantly lower.

Simultaneously, the outcomes that can be caused by a diverse character of interactions will follow the law of events' probability. To illustrate, consider the loss of functionality in the DNA damage repair system. This system's threshold value can be reached after several types of different preceding interactions, leading to its damage. These events may include but are not limited to the loss of the responsible genes' structure, downregulation of these genes by other genes that were transformed, alteration of DNA polymerase functionality, etc. Considering this variability, it is difficult to estimate the exact threshold for this system since several sources of transformations can cause the loss of functionality.

Once the damage occurs and the system no longer provides functionality, it starts producing damage to the whole-cell system, which upon reaching its threshold of damage will become non-functional. Considering this, aging organisms have a certain maximum lifespan until the system reaches a threshold level. Deleterious events from both internal and external factors damage different parts of the system until it reaches the final thresholds, leading to a cell's transformation into a non-functional state. This concept could be applied to any system, and as a result, the prevention of interactions having probabilities to cause thresholds could result in the avoidance of a functional state decay (dead cell, cancer cell, etc.).

### Nature of transformations during aging

To summarize, some of the key principles of particle physics can be borrowed to study biological systems that age. Namely, these principles are:

1. Every interaction leads to a transformation of all interacting subjects.
2. Every process can be dissociated from the chronological time and considered as a sequence of discrete events. It is the order and the number of these events that predetermines the outcome, not time.
3. The threshold value is predetermined by a probability for a specific interaction to cause a specific transformation. An at-the-threshold event occurs not because of the accumulation of prior stimuli but because an increasing number of stimuli increased the probability of the observed event.

These principles can help understand the nature of aging or other biological processes. Cell functionality is determined by complex interactions of atoms within micro- and macromolecules with various cell structures and internal cell environments. During aging, discrete transformations in a cell eventually negatively affect cell system functionality. When atoms within molecules change their characteristics due to interactions and transformations, such as radioactivity, oxidation, reduction, etc., the macromolecule's whole functionality is altered. Alteration of functionality causes direct damage to a system by performing an alternative function or indirect damage defined by a loss of functionality. In other words, systematic damage accumulation from the standpoint of a whole system leads to the decay of the system. Once damage in a system reaches a certain magnitude i.e. reaches a threshold, the system stops performing the originally designated function. When a cell can no longer maintain critical functionality, it finally transforms into a highly non-functional state. On an organism level, outcomes can look differently, e.g., senescence, cancer, coronary diseases, diabetes.

Therefore, biological aging may be defined as a sequence of highly discrete transformations caused by a combination of internal and external factors that lead to chronic damage accumulation and the consequent loss of functionality of a system. The transformations of damage repair mechanisms themselves may lead to their reduced functionality, representing critical thresholds since it would increase the rate of damage accumulation. However, some systems also exist where damage dilution, partitioning, clearance, decay and/or pre-emption support cell rejuvenation, thereby making the biological system appearing as non-aging (e.g. mammalian cell lines, germline, hydra).

### Practical applications of the model

Once the certain functionality stops to be maintained, it means that the damage became too high for a system, and the system consequently reached its threshold after a particular transformation. Analysis of thresholds as discretely as possible, i.e., analyzing the state of a functional unit (e.g., a molecule or a cell) before and after a particular transformation using transcriptomics, proteomics, genomics, and other approaches, could help identify key discrete interactions that led to changed configurations. Isolation of a transformed element and its further analysis to determine the exact cause of transformation can help develop interventions protecting that specific discrete element from a specific type of interaction causing its transformation. Targeting the thresholds to stop, slow, reverse or modulate the direction of inference, represented by the transformations preceding and increasing the probability of a particular outcome (specific pathology, condition, or death) could be an example of an effective longevity intervention. Below, we consider three possible examples of how to identify and affect discrete threshold probabilities, which could help understand aging mechanisms in greater detail and design novel longevity interventions.

### Example 1. Prevention of caspase-mediated neuronal cell death

Different types of cell death, such as apoptosis, necrosis, pyroptosis, ferroptosis and necroptosis, are the processes regulated by internal and external factors and aimed at regulating homeostasis in a living organism. However, the dysregulation of these processes has been implicated in aging [29]. In particular, we would like to recall the example of the programmed cell death in neurons, the rate of which tends to increase with age [30, 31]. Neurological disorders are known as the major reason of elderly population disability and as the second leading cause of death [32]. Therefore, neuroprotective interventions deserve greater attention from researchers in the field.

The major pathways that regulate the neuronal cell death in aging are necrosis (as a response to severe hypoxia) and apoptosis (as a response to other types of damage that do not affect cell membrane integrity) [33, 34]. Both are mediated by caspase-9 that activates caspase-3, a protein that plays a central role in starting the apoptotic process. However, it is important to mention that despite the efficacy of caspase-3 inhibitors in preventing neuronal death [33], this approach is questionable since caspase-3 is also an important mediator of neuronal plasticity and thus is needed for the memory and learning process. As previously shown, whether caspase-3 would lead to apoptosis or plasticity depends on the environmental context (proximity to the synapse, intensity of a signal, length of neuronal activation). Independent from each other, a high magnitude of each of these contextual features equally leads to the programmed cell death while low magnitude, on the contrary, enhances plasticity [34]. Therefore, the cell survives while the magnitude of the environment is below a certain threshold level. As an organism ages, the cumulative number of neurons that meet these apoptosis requirements increases as this pathway is a probability density-dependent outcome representing an example of a threshold. Based on the result, it is likely that a caspase-3 inhibitor is not an optimal solution for preventing neuronal death (and its consequences in the form of neurodegenerative diseases) because the inhibition of caspase-3 would also lead to a reduced ability of neurons to adapt.

To summarize, specific sequences of pathways lead to certain caspase-3 mediated outcomes in these cases. In the case of apoptosis, caspase-9 was reported to be activated by cytochrome c as a response to damage accumulation, but in the case of plasticity, the mechanism is different and is yet to be understood [33, 34]. The negative role of caspase-9 for learning and memory supports the idea that the mechanisms leading to enhanced plasticity and apoptosis are distinct and have different types of thresholds [35]. Moreover, reduction of caspase-9 activity was shown to have neuroprotective activity as a therapeutic approach to treat the development of dementia. Therefore, inhibition of caspase-9 in neurons after middle-age seems to be an attractive longevity intervention to be tested. Some preliminary results show that inhibition of caspases rescues neurons from death [33, 36, 37], but the effect of this intervention on longevity and or/neurodegeneration in aging has not been tested yet. This therapy's limitation is the relatively high cost of specific caspase inhibitors; however, recently, it was shown that the commonly used non-steroid anti-inflammatory drug ibuprofen acts as a caspase enzyme family inhibitor [38]. It was shown that ibuprofen increases the lifespan of model organisms, such as *S. cerevisiae*, *C. elegans* and *D. melanogaster* [39]. Ibuprofen was proven to have geroprotective and radioprotective activity, inducing resistance of *D. melanogaster* to oxidative and genotoxic stress conditions, sufficiently activating DNA repair and heat shock response genes, and positively affecting the median lifespan of flies when being introduced after middle-age [40]. Finally, long-term ibuprofen treatment of mice, with genetically enhanced NF-kB activity (i.e. having a chronic low-grade inflammation), resulted in reduced neuroinflammation and senescence markers in mice neurons as well as in improvements of memory capacity and cognitive function [41].

### Example 2. Understanding neuronal rejuvenation

It is considered as an axiom that differentiated neurons do not undergo mitotic division, meaning that neurons do not dilute the accumulated damage by this mechanism [42, 43]. However, brain tissue ages similarly to other tissues, including those with proliferative potential. Moreover, the cerebellum ages slower than other parts of the human body [44]. This example of neurons (and by analogy, other non-dividing non-replaceable cells) suggests that when dilution of damage by cell division is not available to slow the aging process, other designated molecular mechanisms for clearance of damage evolved. One possible protection mechanism for neuronal damage is drainage of the damage into the glymphatic system during sleep [45–49]. Sleep is one of the best examples of thresholds present in the human organism – after a certain amount of activity, an organism can no longer maintain its functionality. Sleep deprivation negatively affects a wide range of emotional and cognitive functionality, including, but not being limited to working memory, attention, reward processing, aversive stimulus processing, and hippocampal memory processing [50]. It was found that, during sleep, neurons transform into a shrunken form, resulting in an increase of interstitial fluid space by 60%. During this process, neurons expel part of their metabolome into the interstitial fluid, which results in an increased metabolome exchange between interstitial fluid and cerebrospinal fluid. This, in turn, leads to enhanced clearance of toxic substances and waste products through the glymphatic system. After that, the “filtered” metabolome is absorbed back into the neuron. Basically, neurons could exist in 2 major configurations – awake and react or asleep and recover [46]. It does not mean to be absolute – during sleep, neurons can still react, and during awareness, neurons still undergo recovery by expelling toxic substances and byproducts into the glymphatic system, but this process is 95% slower [47]. Impairments of the sleep drainage system are observed in aged organisms [51].

Explanation of this phenomenon from the perspective of the threshold principle is that once the threshold value for the damage inside the neuron is reached, the probability of activation of a pathway leading to a transformation of the neuron into the asleep configuration becomes very high. As a result, sleep is needed for recovery, and neurons clear damage accumulated in them. It was shown that acute and chronic sleep deprivation results in epigenetic changes and, consequently, brain cognitive and emotional functionality impairments [52–54]. Sleep deprivation results in an increased speed of DNA methylation [55], alterations of transcriptional and epigenetic profiles of circadian clock genes [56], induction of oxidative stress and ATP depletion [57]. Insomnia is associated with higher epigenetic age of blood, suggesting that insomnia results in accelerated aging of an organism [58]. However, mechanisms of neuronal rejuvenation during sleep have not been studied in detail.

Based on this, transcriptome, proteome, and methylation state of a neuron before, during, and after sleep could be analyzed to answer the following questions:

1. What is the specific system that senses the damage and signals resulting in a need for sleep?
2. Could the same pathway be activated in other body cells to induce damage clearance?
3. What is the effect of sleep on the rejuvenation of a neuron?

Moreover, there is a potential to develop a novel technique for neuron rejuvenation. As a first step, using an appropriate cell line (e.g., PC12 rat cell line) young neurons may be grown. They may be used as a source of the “young” metabolome, which could be injected into an old rat's glymphatic system during sleep. Finally, the transcriptome, proteome, and DNA methylation state of neurons before and after the procedure may be analyzed to determine how the young metabolome affects old neurons.

### Example 3. Follicle as a short-lived human cell model

Women’s menstrual cycle is another example where human aging may be analyzed by using the principles learned from particle physics. From day 1 to approximately day 14 of the menstrual cycle, the ovarian follicle grows and develops to rupture and free the oocyte (ovulation). As soon as the oocyte leaves the follicle, a transformation occurs, and the follicle becomes the corpus luteum, a temporary endocrine structure. As the oocyte travels to the uterus, the corpus luteum produces progesterone, which is essential for the oocyte to attach to the uterus and for the endometrium decidualization. Further, depending on the fate of the oocyte, corpus luteum can further undergo two possible alternative configurations. If fertilization does not occur after ~nine days from the moment of follicle rupture, the corpus luteum will decay by apoptosis and stop producing progesterone, which will result in menstruation. However, if fertilization occurs, the trophoblast cells will produce human chorionic gonadotropin (HCG). This hormone does not let the corpus luteum decay until the womb itself can produce enough progesterone (10 weeks since impregnation) [59]. So, depending on chorionic gonadotropin presence or absence only, the system can live a very short or 10-fold longer life.

Considering this case from the perspective of particle physics’ principles:

- The menstrual cycle could take from 26 to 31 days of chronological time. It occurs as a sequence of specific transformations, one triggering another.
- There are several examples of thresholds leading to different configurations for the follicle. The first one happens when the oocyte leaves the follicle and triggers the corpus luteum to produce progesterone. Second, in the presence of HCG, the cell transforms from a short-lived to a longer-lived cell. The third is an event leading to apoptosis of the corpus luteum.

While it is not fully understood why the decay of the corpus luteum in the absence of HCG occurs, an analysis of existing data allows us to hypothesize a possible mechanism. In the state of active progesterone production, the corpus luteum produces inhibin, a glycoprotein hormone, with one of the main functions being follicle-stimulating hormone production inhibition. It was shown that in mutant follicles with repressed inhibin α gene expression (30%–40% decrease in production), the apoptosis index was significantly higher compared to normal follicles [60]. At the same time, in another study, it was found that in the luteal phase, the genome of the corpus luteum goes through enhanced methylation, specifically at the inhibin α gene promoter region [61]. The methylation blocks the access to relevant transcription factors leading to the reduction of inhibin production. Histone modification precedes enhanced methylation at H3K9 and H3K27, and the authors assume that these changes facilitate the enhanced methylation. Together, these two studies suggest that enhanced methylation and the gradual reduction of inhibin biosynthesis trigger the corpus luteum to initiate apoptosis, while the presence of HCG counteracts this threshold.

To the best of our knowledge, this process has never been considered in the context of aging. Understanding the mechanisms occurring in the follicle during the fertilization/survival and no fertilization/apoptosis scenarios would allow us to study these thresholds in the context of aging. Moreover, studying the effect of inhibin on the survival of other cell types and model organisms could explain its mechanisms of action and potentially lead to the development of a novel intervention with a similar mode of action. The data showing that inhibin levels decrease with age [62, 63] and that follicle-stimulating hormone levels increase with age contributing to the process of aging [64] indicates that the role of inhibin in aging could have been overlooked.

## CONCLUSIONS

There is an agreement that aging research as a field lacks a common ground that would allow reaching consensus at least in some aspects of the nature of the aging process. While most unifying aging theories are based on inductive reasoning, we applied deductive reasoning to find aging principles that could satisfy different theories’ advocates. The deductive reasoning is here based on the particle physics’ principles. Three principles that we found to be most applicable for describing aging as a process are interaction-transformation, discreteness, and threshold. Applying these principles, we can describe aging as a sequence of highly discrete transformations caused by a combination of internal and external factors that lead to chronic damage accumulation and the consequent loss of functionality of a system after reaching a certain threshold, resulting in the transformation of a young functional organism into an aged non-functional one. This definition is in line with most existing theories of aging. Considering the complexity of a living system that ages, an uncountable number of deleterious events occur throughout the lifetime. The combination of these events results in the accumulation of the deleteriome - cumulative damage of genetic, environmental, and natural physicochemical origin happening at every level of functionality, leading to the loss of the organism’s functionality.

These principles suggest that effective anti-aging interventions would be the tools that postpone or abolish the thresholds for specific transformations at discrete levels, or methodologies, leading to slowing down the rate of transformation of a system from a functional to damaged state or to the transformation of a system from a damaged to a functional state.
